# Perinatal Nitric Oxide Therapy Prevents Adverse Effects of Perinatal Hypoxia on the Adult Pulmonary Circulation

**DOI:** 10.1155/2014/949361

**Published:** 2014-07-08

**Authors:** Anne-Christine Peyter, Flavien Delhaes, Giacomo Diaceri, Steeve Menétrey, Jean-François Tolsa

**Affiliations:** Neonatal Research Laboratory, Clinic of Neonatology, Department of Pediatrics, Lausanne University Hospital, 1011 Lausanne, Switzerland

## Abstract

Adverse events in utero are associated with the occurrence of chronic diseases in adulthood. We previously demonstrated in mice that perinatal hypoxia resulted in altered pulmonary circulation in adulthood, with a decreased endothelium-dependent relaxation of pulmonary arteries, associated with long-term alterations in the nitric oxide (NO)/cyclic GMP pathway. The present study investigated whether inhaled NO (iNO) administered simultaneously to perinatal hypoxia could have potential beneficial effects on the adult pulmonary circulation. Indeed, iNO is the therapy of choice in humans presenting neonatal pulmonary hypertension. Long-term effects of neonatal iNO therapy on adult pulmonary circulation have not yet been investigated. Pregnant mice were placed in hypoxia (13% O_2_) with simultaneous administration of iNO 5 days before delivery until 5 days after birth. Pups were then raised in normoxia until adulthood. Perinatal iNO administration completely restored acetylcholine-induced relaxation, as well as endothelial nitric oxide synthase protein content, in isolated pulmonary arteries of adult mice born in hypoxia. Right ventricular hypertrophy observed in old mice born in hypoxia compared to controls was also prevented by perinatal iNO treatment. Therefore, simultaneous administration of iNO during perinatal hypoxic exposure seems able to prevent adverse effects of perinatal hypoxia on the adult pulmonary circulation.

## 1. Introduction

Numerous epidemiological studies have associated adverse events occurring in utero, like limitation of nutrients or oxygen supply, with an increased risk to develop chronic diseases in adulthood, including coronary artery disease, systemic hypertension, stroke, and noninsulin-dependent diabetes mellitus [1]. Therefore, an insult occurring in the perinatal period, namely, during late gestation and the first days of extrauterine life, may result in a definitive imprint predisposing to a pathological response later in life, leading to “programmed” diseases. However, cellular and molecular mechanisms of the programming process are not yet elucidated [2, 3]. In particular, chronic pulmonary vascular diseases and abnormal pulmonary vascular reactivity in adulthood may be associated with a hypoxic insult occurring around birth [4–8]. Humans and animals born in hypoxic conditions, or presenting neonatal pulmonary hypertension, show later in life an exaggerated pulmonary hypertensive response following a reexposure to hypoxia [7, 9–13]. The cellular and molecular mechanisms involved in the dysregulation of adult pulmonary vasomotor tone secondary to a transient perinatal insult remain incompletely understood.

The fetal pulmonary circulation is characterized by low perfusion and high vascular resistances, which need to rapidly fall at birth to allow pulmonary blood flow to increase nearly 10-fold [14, 15]. Pulmonary vascular tone is regulated by a complex and intricate group of mechanisms [16, 17], including the nitric oxide/cyclic guanosine monophosphate (NO/cGMP) signaling pathway, which plays a key role in establishing relaxation of the pulmonary vessels during neonatal transition as well as later in life [18, 19]. Sometimes, the normal decrease in pulmonary vascular resistances and increase in pulmonary blood flow do not occur, resulting in persistent pulmonary hypertension of the newborn (PPHN). PPHN is a severe neonatal complication, associated with a high mortality and morbidity. Prevalence of PPHN is estimated at 1.9 per 1000 live births and mortality ranges from 4 to 33% [20]. This pathology represents 1 to 4% of the admissions in a neonatal intensive care unit [21, 22] and requires intensive therapy including supplemental oxygen and pulmonary vasodilators [23]. Inhaled NO (iNO) has been proposed as a “selective” pulmonary vasodilator since it acts directly on pulmonary vascular cells and is then inactivated in the bloodstream. Inhaled NO is now recognized as the therapy of choice for term newborns with a PPHN [24]. Most neonates who respond to this treatment will recover within a week and then develop normally. However, long-term effects of neonatal iNO therapy on adult lung vascular reactivity have not yet been investigated.

Adult pulmonary hypertension is also a severe disease of various origins, affecting life expectancy and quality of life [25], whose treatment is still a challenge and prognosis remains poor [26]. Pulmonary hypertension is related to an abnormal reactivity of the pulmonary vasculature resulting in lower perfusion of the lung and hypoxemia. Abnormal development of regulatory mechanisms of the pulmonary circulation in the perinatal period could be associated with an altered regulation of the adult pulmonary circulation.

We have previously established a murine experimental model in order to study the long-lasting effects of a transient exposure to hypoxia in the perinatal period [13, 27–29]. Pregnant mice were placed in hypoxic conditions (13% O_2_) 5 days before delivery and left with their litter for 5 days after birth. Pups were then bred in normoxia until adulthood when studied. We first demonstrated that the relaxation induced by the endothelium-dependent agent acetylcholine (ACh) in the adult main pulmonary artery (PA) was completely mediated by endothelial nitric oxide synthase (eNOS) [28]. Our further studies showed that a transient hypoxic insult in the perinatal period resulted in altered regulation of pulmonary vascular tone in adulthood [13, 27, 29]. Adults born in hypoxia displayed an increase in right ventricular pressure, suggesting a higher resistance state in the pulmonary circulation, and an increased sensitivity to acute hypoxia compared to controls [13]. Moreover, perinatal hypoxia dramatically decreased endothelium-dependent relaxation induced by ACh in adult female pulmonary arteries [13]. This alteration of adult pulmonary circulation was associated with long-term alterations in the NO/cGMP signaling pathway, in particular muscarinic receptors and phosphodiesterases, and with a marked reduction in eNOS protein content in isolated pulmonary arteries from adult females born in hypoxic conditions compared to mice born in normoxia [13]. This could contribute to the observed decrease in ACh-mediated relaxation following perinatal hypoxia.

As iNO is actually the therapy of choice in humans presenting neonatal pulmonary hypertension, we decided to investigate in our murine experimental model whether iNO administered simultaneously to perinatal hypoxia could have potential beneficial effects on the adult pulmonary circulation.

Effects of concomitant perinatal exposure to iNO and hypoxia were compared to those of perinatal hypoxia alone by investigation of ACh-induced relaxation and eNOS protein content in isolated pulmonary arteries, completed by anatomical data in young and old adult females.

In our murine experimental model, perinatal hypoxia triggered greater alterations in endothelium-dependent relaxation and the NO/cGMP pathway in adult females than adult males (unpublished data). Our previous manuscript [13] was therefore focused on alterations occurring in the pulmonary circulation of adult females. The present report was consequently also limited to the effects of iNO in females. The results obtained in mice exposed to perinatal hypoxia and iNO were directly compared to the corresponding data we previously published for mice born in normoxia or hypoxia alone [13], in particular for ACh-induced relaxation and anatomical data. Indeed, the present study was the direct continuation of our previous work in which we used exactly the same experimental model [13]. As all procedures were similar between both studies, we did not repeat the experiments performed in mice born in normoxia or hypoxia alone and therefore some data of the referred article [13] are shown in the present paper to better highlight the beneficial effects of iNO therapy compared to hypoxia alone.

## 2. Methods

### 2.1. Murine Model of Perinatal Hypoxia

**Figure 1:**
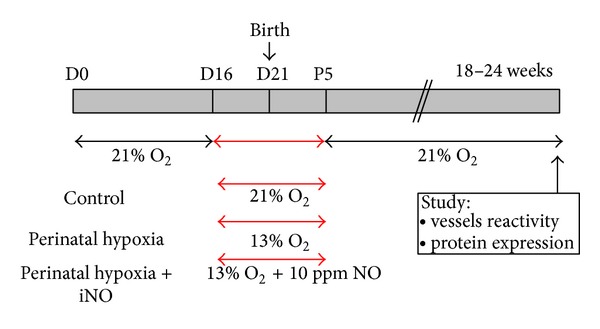
Experimental design. C57BL/6 pregnant mice were exposed from 5 days before delivery until 5 days after birth either to normoxia, hypoxia, or hypoxia with simultaneous administration of inhaled NO (iNO). Pups were then raised in normoxia until adulthood. Mice were studied as young adults (5-6 months) or old adults (12–15 months).

All experimental procedures were approved and carried out in accordance with the Swiss Veterinarian Animal Care Office. C57BL/6 pregnant mice were purchased from Harlan (Horst, The Netherlands). They were all fed* ad libitum* and exposed to day-night cycles. Perinatal hypoxia was induced as previously described [13, 27] (Figure 1). Pregnant mice were placed in hypoxic conditions (13% O_2_) 5 days before delivery and left under hypoxia with their litter for 5 days after birth. Pups were then raised in normoxia (21% O_2_) until adulthood. Pups born and grown in normoxic conditions were used as controls. Female mice were studied as adults before week 25 (young females) or after week 50 (old females).

### 2.2. Perinatal Exposure to iNO

Pregnant mice were placed in an atmosphere containing 13% O_2_ and 10 ppm gaseous NO for 10 days, more exactly for 5 days before delivery and 5 days after birth (Figure 1). Pups were then raised in normoxia (21% O_2_) until adulthood when studied.

Inhaled NO was administered using the SONIMIX apparatus (LNIndustries, Geneva, Switzerland), which has been adapted to allow injection of gaseous mixtures containing oxygen, nitrogen, and NO according to the preset relative concentrations. The applied concentration of gaseous NO was half the concentration currently used in clinical practice to treat human newborns with pulmonary hypertension. NO concentration in the box was monitored using a NO sensor (SensorNox, SensorMedics).

### 2.3. Anatomical Data

Body weight was measured 5 days after birth. Number of alive pups was recorded at the end of the perinatal exposure to normoxia, hypoxia, or hypoxia plus iNO.

In adults, the heart was dissected with removal of the auricles and separation of the right ventricle (RV) and the left ventricle plus septum (LV + S). The RV/(LV + S) ratio was calculated and used as an index of right ventricular hypertrophy, which is a direct consequence of increased pulmonary vascular resistances present in pulmonary vascular pathologies.

### 2.4. Vascular Reactivity Studies

The vascular reactivity of pulmonary arteries was investigated by isolated vessel tension studies, as previously described [13]. Briefly, adult mice were administered a lethal dose of pentobarbital (1 g/kg intraperitoneal) and the main pulmonary artery was immediately harvested. The vessel ring was suspended in organ chamber filled with 10 mL of modified Krebs-Ringer bicarbonate solution (mM: 118.3 NaCl, 4.7 KCl, 2.5 CaCl_2_, 1.2 MgSO_4_, 1.2 KH_2_PO_4_, 25.0 NaHCO_3_, and 11.1 glucose) maintained at 37°C and aerated with 95% O_2_-5% CO_2_ (pH 7.4) [13, 27]. Vessels were brought to their optimal resting tension after 2 stretches of 0.5 g. After equilibration, indomethacin (10^−5^ M) was added in order to exclude possible interference of endogenous prostanoids. The vessels were then contracted with phenylephrine (10^−5^ M) and pharmacological response of isolated pulmonary arteries was evaluated in the presence of increasing concentrations of the endothelium-dependent relaxing agent ACh. Change in tension induced by the vasodilator was expressed as percent of the initial contraction induced by phenylephrine.

### 2.5. Western Blotting Analysis of Protein Expression

Specific protein content in pulmonary arteries was investigated by western blotting analysis as previously described [13]. Briefly, each flash-frozen main PA was crushed in a cryogenic mortar and homogenized in 26 *μ*L of lysis buffer (50 mM HEPES, 1 mM EDTA, 1 mM EGTA, 10% glycerol, 1 mM DTT, 5 *μ*g/mL pepstatin, 3 *μ*g/mL aprotinin, 10 *μ*g/mL leupeptin, 0.1 mM 4-(2-aminoethyl)benzenesulfonyl fluoride hydrochloride, 1 mM sodium vanadate, 50 mM NaF, and 20 mM 3-[(3-cholamidopropyl)dimethylammonio]-1-propanesulfonate). After a 45 min incubation on ice, the homogenates were centrifuged for 10 min at 3000 ×g at 4°C. Five *μ*L of each supernatant was diluted in Laemmli buffer and heated for 5 min at 95°C before loading on a 7.5% polyacrylamide gel. Proteins were fractioned by SDS-PAGE (35 min at 200 V) and transferred to PVDF membrane (Bio-Rad, Hercules, CA, USA) during 2 h at 100V. Blots were blocked overnight at 4°C in Tris-buffered saline plus 0.05% Tween 20 (TBS-T) containing 5% nonfat dry milk. All washing steps were performed using TBS-T. Membranes were immunoblotted for 1 h at room temperature using specific antibodies targeted against eNOS (BD Transduction Laboratories, Franklin Lake, NJ, USA), or *β*-actin (Sigma), diluted 1 : 200 (for eNOS), or 1 : 250 (for *β*-actin) in TBS-T containing 5% nonfat dry milk. Blots were then incubated with ECL horseradish peroxidase-linked anti-mouse antibody (for eNOS detection) or anti-rabbit antibody (for *β*-actin) (Amersham Biosciences, Buckinghamshire, UK), diluted 1 : 10,000 in TBS-T. Finally, specific proteins were detected by chemiluminescence using the SuperSignal West Pico Chemiluminescent Substrate (Pierce) and exposition to X-ray film. Expression of eNOS was quantified using the UN-SCAN-IT gel software (Silk Scientific, Orem, UT, USA) and normalized to *β*-actin content. Relative protein content in homogenates of mice born in hypoxia was reported to the content measured in controls.

### 2.6. Drugs

Unless otherwise specified, all drugs were purchased from Sigma (St. Louis, MO). Indomethacin was prepared in equal molar Na_2_CO_3_ [30]. The other drugs were prepared using distilled water.

### 2.7. Data Analyses

Statistical analyses were performed using InStat 3.0 or Prism 4.0 (GraphPad Software, San Diego, CA). Data are expressed as mean ± SEM, and *n* represents the number of animals per group. Unless otherwise mentioned, the influence of perinatal exposure to hypoxia and/or iNO on the studied parameters was analyzed using the Mann-Whitney test. Two-way ANOVA was performed to compare the dose-response curves in isolated vessel tension studies. The difference was considered statistically significant when *P* < 0.05.

## 3. Results

### 3.1. Neonatal Data

Table 1 summarizes body weight and number of pups per litter recorded at the end of the 10-day exposure to normoxia, hypoxia, or hypoxia plus iNO. Body weight was significantly reduced in 5-day-old pups born in hypoxia, with or without iNO, compared to controls. The number of alive pups per litter was also significantly lower after perinatal exposure to hypoxia, in the absence or presence of iNO, compared to normoxia. Although the number of alive pups appeared to be higher in litters exposed to perinatal hypoxia and iNO than in those with perinatal hypoxia alone, the difference was not statistically significant.

### 3.2. Anatomical Data in Adults

Table 2 summarizes anatomical data measured in adult females after sacrifice.

In young females (5-6-month-old mice), perinatal hypoxia did not influence the studied parameters, as previously published [13]. Simultaneous exposure to perinatal hypoxia and iNO, however, significantly reduced heart weight, the heart to body ratio, and RV weight compared to mice born in normoxia or hypoxia alone. The LV + S weight was also decreased in mice exposed to hypoxia plus iNO compared to controls. Finally, the RV/(LV + S) ratio seemed lower in mice exposed to hypoxia and iNO than in controls or mice with perinatal hypoxia, but the difference was not quite significant between mice born in hypoxia in the presence or absence of iNO (*P* = 0.0677, using the Mann-Whitney test), as well as between mice born in hypoxia plus iNO and controls (*P* = 0.0967, using the Mann-Whitney test).

In old females (12–15-month-old mice), anatomical data were similar between mice exposed to perinatal hypoxia and controls, except for the RV/(LV + S) ratio, which was significantly higher in mice born in hypoxia than in controls. Old females submitted to simultaneous exposure to perinatal hypoxia and iNO displayed similar anatomical data to controls. However, RV weight and the RV/(LV + S) ratio were significantly reduced compared to mice exposed to perinatal hypoxia alone.

### 3.3. Isolated Pulmonary Artery Reactivity

**Figure 2:**
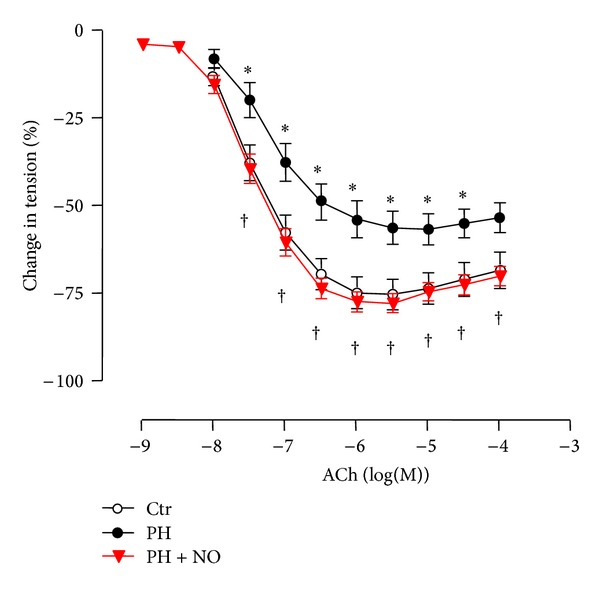
Pharmacological response of isolated pulmonary arteries to the endothelium-dependent agent acetylcholine. Dose-response to acetylcholine (ACh) was tested in pulmonary arteries preconstricted with phenylephrine (10^−5^ M), isolated from adult mice born in normoxia (Ctr), in hypoxia (PH), or in hypoxia with simultaneous administration of iNO (PH + NO). Results are expressed as mean ± SEM of percentage of change in tension induced by the vasodilator (*n* = 6–9 mice). *Significant difference compared to controls and ^†^significant difference between mice exposed to perinatal hypoxia and iNO and mice with perinatal hypoxia alone (*P* < 0.05, two-way ANOVA). Results obtained with pulmonary arteries of mice born in normoxia (Ctr) or in hypoxia (PH) were previously published [13].

Pharmacological response to cumulative doses of the endothelium-dependent relaxing agent acetylcholine (ACh) was tested in isolated PA preconstricted with phenylephrine (Figure 2). We previously showed that ACh-induced relaxation was dramatically reduced in isolated PA from adult females born in hypoxia compared to controls [13]. In contrast, isolated PA from adult mice exposed simultaneously to hypoxia and iNO in the perinatal period displayed a dose-dependent relaxant response to ACh, which was similar to controls. Therefore, ACh-induced relaxation was significantly increased in mice exposed to perinatal hypoxia plus iNO compared to perinatal hypoxia alone (Figure 2).

### 3.4. eNOS Protein Content

**Figure 3:**
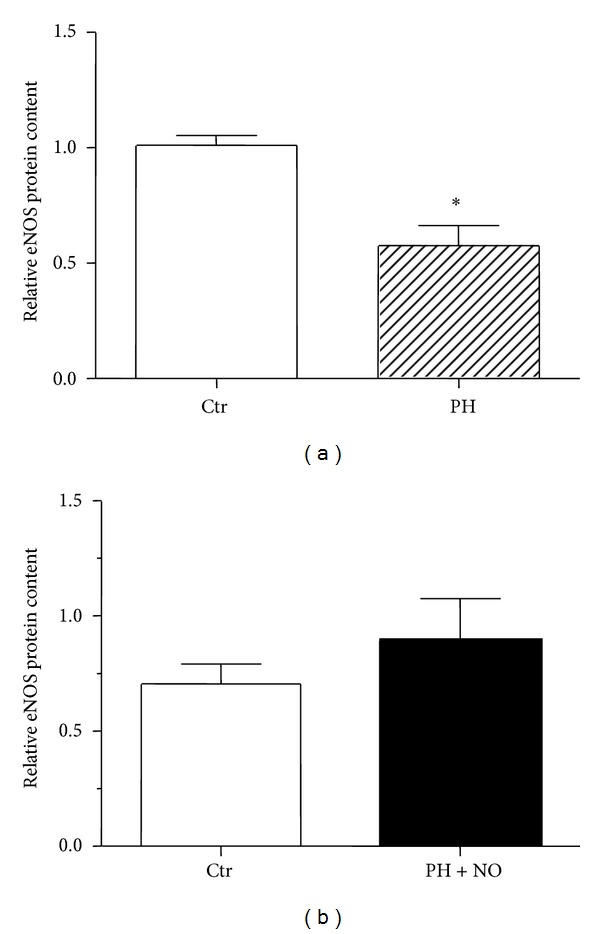
Western blotting analysis of eNOS protein expression in pulmonary arteries. (a) Western blotting analysis of eNOS protein relative content was performed in pulmonary arteries extracts (*n* = 3 pools of 10 PA) of adult females born in normoxia (Ctr) or in hypoxia (PH). These data were previously published in [13]. *Significant difference between Ctr and PH groups (*P* < 0.05 using unpaired *t*-test). (b) Relative eNOS protein content was analyzed in pulmonary arteries extracts of adult mice born in normoxia (Ctr) or exposed simultaneously to hypoxia and iNO in the perinatal period (PH + NO) (*n* = 4 mice). Results are expressed as mean ± SEM of the relative eNOS protein content after normalization by *β*-actin protein content.

We previously showed that the eNOS protein relative content was significantly reduced in the main PA of adult females born in hypoxia compared to controls [13] (Figure 3(a)). In contrast, the eNOS protein relative content in PA of adult females exposed simultaneously to perinatal hypoxia and iNO was similar to controls (Figure 3(b)).

## 4. Discussion

We previously demonstrated, in a murine model of perinatal hypoxia, that a transient exposure to hypoxia during a critical period of development induced long-term adverse effects on the adult pulmonary circulation [13, 27, 29].

In the present study, we investigated in the same experimental model whether iNO administration during the whole exposure to perinatal hypoxia could have beneficial effects on the adult pulmonary circulation. Potential protective effects of iNO were tested by measurement of several parameters which were found to be altered in adult females exposed to perinatal hypoxia [13].

In our previous report [13], we demonstrated that perinatal hypoxia induced a marked decrease in endothelium-dependent ACh-induced relaxation of PA, whereas no difference was found in the relaxation induced by gaseous NO or NO donors. As the present study aimed to investigate potential protective effects of iNO against adverse effects of perinatal hypoxia, the functional experiments were therefore limited to the pharmacological response of isolated PA to ACh, which was altered in adult mice exposed to perinatal hypoxia alone compared to controls, whereas relaxation induced by the NO donor 2-(N,N-diethylamino)-diazenolate-2-oxide (DEA/NO) was similar in mice born in hypoxia and controls.

Vascular reactivity studies showed that although ACh-induced relaxation was significantly reduced in isolated PA from adult females exposed to perinatal hypoxia compared to controls, simultaneous administration of iNO during perinatal hypoxic exposure completely restored the vasorelaxant response to ACh.

Similarly, perinatal exposure to hypoxia with concomitant iNO treatment allowed a total recovery in eNOS protein content in the main PA of adult females, whereas eNOS protein expression was shown to be reduced by almost 40% in adult females born in hypoxia compared to controls [13].

Anatomical data recorded in young adult mice (5-6 months) were similar between females born in hypoxic conditions and controls. Although previous hemodynamic studies showed a higher right ventricular pressure in 5-month-old mice exposed to perinatal hypoxia compared to controls [13], strongly suggesting a higher resistance state in the pulmonary circulation, the RV/(LV + S) ratio was not significantly increased in young adult females. The RV/(LV + S) ratio was used as an index of right ventricular hypertrophy, which is a direct consequence of increased pulmonary vascular resistances accompanying pulmonary hypertension. Here we showed that, in old females (12–15 months), the RV/(LV + S) ratio was significantly higher in mice born in hypoxia than in controls, suggesting that perinatal hypoxia resulted in right ventricular hypertrophy later in life, probably as a result of increased pulmonary vascular resistances following perinatal exposure to hypoxia. Inhaled NO treatment during perinatal hypoxia significantly reduced RV weight compared to mice exposed to perinatal hypoxia alone, both in young and old females. In old females exposed to perinatal hypoxia and iNO, the RV/(LV + S) ratio was also significantly decreased compared to mice born in hypoxia alone and thus became similar to the RV/(LV + S) ratio observed in controls.

Taken together our results suggest that concomitant administration of iNO during the whole exposure to perinatal hypoxia exerts a protective effect on the pulmonary circulation. Such results argue in favor of a potential long-term beneficial effect of neonatal iNO therapy. It will be nevertheless necessary to investigate whether neonatal (instead of perinatal) treatment would confer the same protection and whether this protective effect could also be observed on additional parameters, like right ventricular pressure, sensitivity to acute hypoxia, and molecular components of the NO/cGMP pathway, such as muscarinic receptors and phosphodiesterases, which were found to be altered in adult females exposed to perinatal hypoxia [13].

Beneficial effects of iNO were also shown in other experimental models. For example, a 10-day treatment with iNO (10 ppm) during recovery from a 10-day exposure to neonatal hypoxia restored lung structure in eNOS-deficient mice, by stimulation of alveolar and vascular growth [31]. Similarly, concomitant exposure of 9-day-old rat pups to iNO (20 ppm) and hypoxia during 14 days attenuated pulmonary vascular structural changes, right ventricular hypertrophy, and growth retardation induced by exposure to neonatal hypoxia alone [32]. In a model of bleomycin-induced bronchopulmonary dysplasia in neonatal rats, iNO treatment improved lung structure and prevented right ventricular hypertrophy and pulmonary vascular remodeling [33]. In adult rats, continuous inhalation of NO (10 ppm) during a 2-week chronic exposure to hypoxia also reduced pulmonary vascular remodeling and right ventricular hypertrophy compared to rats exposed to hypoxia alone [34]. These reports pointed however mainly to protective effects of iNO against structural pulmonary alterations resulting from hypoxic exposure. More recently, inhalation of NO was found to have cardioprotective effects in murine models of cardiac ischemia-reperfusion injury [35] and to improve outcomes after cardiac arrest and successful cardiopulmonary resuscitation in adult mice [36], even in eNOS-deficient mice [37]. The latter protective effects required the presence of soluble guanylyl cyclase [35, 36]. However, all these studies showed short-term protective effects of iNO administration, whereas our data demonstrate long-lasting beneficial effects of perinatal iNO therapy on the pulmonary circulation throughout life. Indeed, in our experimental model, perinatal iNO administration was able to prevent not only the impairment of endothelium-dependent relaxation in young adults secondary to a perinatal exposure to hypoxia, but also progressive development of right ventricular hypertension in old mice.

The mechanisms implicated in the observed protective effects of iNO treatment during the whole exposure to perinatal hypoxia remain to be elucidated. It will be first necessary to distinguish whether protective effects are mainly due to prenatal or postnatal treatment with iNO.

Prenatal treatment with iNO probably mainly acts through reduction of pulmonary vascular resistances in pregnant mice. Indeed, closed-chest hemodynamic studies showed that acute exposure to iNO in hypoxic conditions was able to reverse hypoxia-induced increase in right ventricular pressure in anaesthetized adult mice (unpublished data). Due to the short half-life of gaseous NO, maternal exposure to iNO during hypoxia may not directly influence the fetal circulation, although there was recent evidence that extrapulmonary effects of iNO could be mediated, in addition to diffusion, by conversion of iNO into S-nitrosothiols further exported into the circulation by the type L amino acid transporter [38]. However, improvement of maternal pulmonary circulation, by reversing hypoxic pulmonary vasoconstriction, could enhance fetal oxygen delivery and perhaps also improve fetal growth. Indeed, placental insufficiency, a pathology associated with decreased nutrients and oxygen supply to the fetus, results in intrauterine growth restriction.

In our murine model of perinatal hypoxia, body weight measured in 5-day-old pups was decreased by about 10% after perinatal hypoxia compared to normoxia. Body weight in pups exposed to perinatal hypoxia plus iNO was also significantly reduced compared to controls and did not significantly differ from pups with perinatal hypoxia alone. This suggests that maternal exposure to iNO did not prevent growth restriction resulting from perinatal hypoxia. Therefore neonatal exposure to iNO during perinatal hypoxia probably contributes largely to the protective effects of iNO against adverse effects of perinatal hypoxia on the adult pulmonary circulation.

Beneficial effects of neonatal iNO therapy could be explained by direct effect on the pulmonary circulation of pups exposed to hypoxia, thanks to the vasorelaxant properties of iNO allowing the counteraction of pulmonary vasoconstriction induced by hypoxia. Moreover, if prenatal exposure to hypoxia induced altered eNOS protein expression, exogenous NO could also act by compensation for endogenous NO production deficiency, allowing recovery of NO-mediated relaxation in pups, thus preventing further alterations in pulmonary vascular tone regulation. Such protective effects could be due to prevention of epigenetic modifications in pulmonary vessels leading to long-term alterations in the regulation of pulmonary circulation. In this line, it would be particularly of interest to investigate whether iNO could prevent the imbalance in the NO/cGMP pathway in adult pulmonary vasculature following perinatal hypoxia, with particular attention to muscarinic receptors and phosphodiesterases. Such a result would suggest that an appropriate intervention to counteract adverse effects of a hypoxic insult at a critical period of development could prevent long-term adverse effects of hypoxia on adult pulmonary circulation.

It remains therefore to test whether a neonatal iNO treatment, introduced from birth to the end of the 10-day perinatal exposure to hypoxia, will be able to exert similar protective effects against adverse effects of perinatal hypoxia on the adult pulmonary circulation in our murine experimental model.

It will be also necessary to confirm such observations in humans. Indeed, iNO therapy was introduced about 15 years ago to treat neonates with PPHN. However there is no data on the long-term effects of iNO on the regulation of adult pulmonary circulation in humans. For example, it was shown that young adults having recovered from PPHN before introduction of the iNO therapy (children admitted to the neonatal-care unit of the Lausanne University Hospital for Children between 1972 and 1979) displayed exaggerated pulmonary vasoconstrictive response following reexposure to hypoxia, namely, during high-altitude exposure [7]. It will be therefore interesting to investigate whether such augmented pulmonary hypertensive response to hypoxia could be prevented in young adults having suffered from PPHN treated with iNO in the neonatal period. Potential long-term beneficial effects, besides the short-term prognostic improvement, will be useful indications for neonatologists to assess the safety of the applied therapy.

In terms of basic research, our data may contribute to a better understanding of the regulatory mechanisms of the pulmonary circulation in the adult following a perinatal hypoxic exposure with or without iNO therapy. In terms of clinical implications, our studies may help to devise novel therapeutic strategies to prevent and treat adult pulmonary vascular disorders secondary to perinatal hypoxic events. More generally, such results should stimulate researchers to further investigate cellular and molecular mechanisms implicated in fetal programming of adult diseases in order to design novel targeted early therapeutic interventions to prevent long-term adverse effects of perinatal insults.

It should be noted that most results of the present study are related to the main PA. The overall reactivity differs between the large conduit PA and the more distal resistant pulmonary vessels. The total pulmonary vascular resistance depends on the combination of the reactivity of all these vessels, among which large pulmonary arteries have also been shown to play a role [39, 40]. However, the modifications observed in the main PA of mice born in hypoxia compared to controls were already significant. Therefore other alterations could be expected in more distal vessels. In our previous study [13], we displayed that alterations secondary to perinatal hypoxia were found both in the main PA and at the level of more distal vessels: molecular assays were performed in total lung homogenates as well as in PA extracts, to assess whether molecular changes observed in the main PA were consistent with changes occurring in total lung. Similarly, hemodynamic studies indirectly showed the influence of perinatal hypoxia on distal vessels, which contribute to the resulting pulmonary arterial pressure, as estimated by measurement of the right ventricular pressure. In the present paper, some data, like the prevention of right ventricular hypertrophy in old mice exposed to perinatal hypoxia, also suggest that iNO exerts a protective effect against alterations not only of the main pulmonary artery but also of more distal vessels.

Moreover, our experimental model was complex, because it combined pre- and postnatal stimuli. In our previous studies, the timing of exposure to hypoxia was chosen to cover the period of lung vasculogenesis, during which the functional units of gas exchange develop [41]. Further comparison between effects of prenatal or postnatal hypoxic insult would be of interest to better determine the susceptibility window of the lung vasculature. Moreover, although chronic hypoxia was found to modulate maternal milk composition [42], a postnatal exposure to hypoxia will mainly impact directly the pups, whereas the effects of a prenatal insult will also depend on the maternal metabolic environment and body composition. Exposure of pregnant rats or mice to chronic hypoxia is an established model of intrauterine growth restriction [43], although the effects vary depending on oxygen concentration and timing of exposure. Such experimental models led to reduced pups' body weight, with [44] or without [45–47] decrease in litter size compared to controls. In our murine model or perinatal hypoxia, we observed a significant reduction in both pups' body weight and litter size compared to controls. However, these data were recorded at the end of the perinatal exposure (at postnatal day 5), thus resulting from combined pre- and postnatal insult. Moreover, as food intake is sometimes [45, 47], but not always [46], reduced in pregnant animals exposed to hypoxia, such experimental models could introduce a component of undernutrition. Whether some malnutrition occurs in our murine model of perinatal hypoxia remains to be investigated, because maternal weight changes were not recorded.

Regarding the timing of iNO treatment, we aimed in the present study to show whether iNO administration during the whole exposure to hypoxia could reverse adverse effects of perinatal hypoxia on adult pulmonary circulation. Further investigation of potential beneficial effects of separate prenatal or postnatal treatment would be useful to determine the therapeutic window in which iNO administration could provide the greater protective effect. Such information could help to devise novel targeted therapeutic interventions. Particular attention will be paid to the postnatal treatment, which is closer to the current clinical practice.

However, despite several limitations, the present paper points to beneficial effects of perinatal iNO administration against some adverse effects of perinatal hypoxia on the adult pulmonary circulation. Further investigations will of course be necessary to better understand the involved mechanisms. However, although several studies displayed short-term protective effects of iNO administration, our data are the first, to our knowledge, to demonstrate long-lasting beneficial effects of perinatal iNO therapy on the pulmonary circulation throughout the life.

## 5. Conclusions

In the present report, we demonstrated that although a 10-day exposure to hypoxia in the perinatal period resulted in alterations of the adult pulmonary circulation, simultaneous administration of iNO during the whole exposure to perinatal hypoxia completely restored ACh-induced relaxation, as well as eNOS protein content, in isolated PA of adult females. Right ventricular hypertrophy observed in old mice born in hypoxia compared to controls was also prevented by perinatal iNO treatment. Therefore, concomitant administration of iNO during perinatal hypoxic exposure seems able to prevent adverse effects of perinatal hypoxia on the adult pulmonary circulation.

## Figures and Tables

**Table 1:** Body weight and number of alive pups per litter at 5 days after birth.

5-day-old mice	Control *n* = 119	Perinatal hypoxia *n* = 46	Perinatal hypoxia + iNO *n* = 98
Body weight (g)	2.90 ± 0.03	2.60 ± 0.07*	2.72 ± 0.04*
Pups per litter	7.0 (4–10)	4.5 (3–7)*	6.0 (3–7)*

Body weight and number of alive pups per litter were recorded at the end of the perinatal exposure to normoxia (control), hypoxia, or hypoxia and inhaled NO (iNO). Results are expressed as mean ± SEM for body weight and median (range) for the number of pups per litter; *n* corresponds to the number of animals studied in each group. *Significant difference compared to controls (*P* < 0.05, using the nonparametric Mann-Whitney test).

**Table 2:** Anatomical data.

5-6-month-old mice	Control *n* = 25	Perinatal hypoxia *n* = 20	Perinatal hypoxia + iNO *n* = 13
Body weight (g)	22.83 ± 0.39	22.63 ± 0.36	23.68 ± 0.41
Heart weight (g)	0.100 ± 0.002	0.097 ± 0.001	0.093 ± 0.002^∗†^
Heart/body ratio	0.0044 ± 0.0001	0.0043 ± 0.0001	0.0039 ± 0.0001^∗†^
RV weight (g)	0.0230 ± 0.0005	0.0227 ± 0.0006	0.0205 ± 0.0006^∗†^
(LV + S) weight (g)	0.0771 ± 0.0015	0.0745 ± 0.0009	0.0721 ± 0.0013*
Ratio RV/(LV + S)	0.300 ± 0.006	0.304 ± 0.008	0.285 ± 0.010

12–15-month-old mice	Control *n* = 11	Perinatal hypoxia *n* = 15	Perinatal hypoxia + iNO *n* = 17

Body weight (g)	26.84 ± 1.10	28.15 ± 0.92	27.32 ± 0.64
Heart weight (g)	0.107 ± 0.003	0.107 ± 0.002	0.109 ± 0.002
Heart/body ratio	0.0040 ± 0.0002	0.0038 ± 0.0001	0.0040 ± 0.0001
RV weight (g)	0.0231 ± 0.0010	0.0252 ± 0.0004	0.0231 ± 0.0005^†^
(LV + S) weight (g)	0.0835 ± 0.0023	0.0821 ± 0.0018	0.0855 ± 0.0015
Ratio RV/(LV + S)	0.276 ± 0.009	0.309 ± 0.008*	0.271 ± 0.004^†^

Body weight was measured after sacrifice of adult mice. Heart weight corresponds to the sum of the wet weights of the right ventricle (RV) and of the left ventricle plus septum (LV + S). The RV/(LV + S) ratio is the ratio between the weights of RV and LV + S, used as an index of right ventricular hypertrophy. Results are expressed as mean ± SEM; *n* corresponds to the number of animals in each group. *Significant difference compared to controls and ^†^significant difference between mice exposed to perinatal hypoxia plus iNO and mice with perinatal hypoxia alone (*P* < 0.05, using the Mann-Whitney test). Data related to 5-6-month-old mice born in normoxia (Ctr) or in hypoxia (PH) were previously published [13].
